# Dissecting the role of low-complexity regions in the evolution of vertebrate proteins

**DOI:** 10.1186/1471-2148-12-155

**Published:** 2012-08-24

**Authors:** Núria Radó-Trilla, MMar Albà

**Affiliations:** 1 Evolutionary Genomics Group, Research Programme on Biomedical Informatics (GRIB) - IMIM (Hospital del Mar Research Institute), Universitat Pompeu Fabra (UPF), Dr. Aiguader 88, Barcelona 08003, Spain; 2 Catalan Institution for Research and Advanced Studies (ICREA), Barcelona, Spain

**Keywords:** Low-complexity region, Simple sequence, Amino acid tandem repeat, Vertebrate protein, Slippage

## Abstract

**Background:**

Low-complexity regions (LCRs) in proteins are tracts that are highly enriched in one or a few amino acids. Given their high abundance, and their capacity to expand in relatively short periods of time through replication slippage, they can greatly contribute to increase protein sequence space and generate novel protein functions. However, little is known about the global impact of LCRs on protein evolution.

**Results:**

We have traced back the evolutionary history of 2,802 LCRs from a large set of homologous protein families from *H.sapiens*, *M.musculus*, *G.gallus*, *D.rerio* and *C.intestinalis*. Transcriptional factors and other regulatory functions are overrepresented in proteins containing LCRs. We have found that the gain of novel LCRs is frequently associated with repeat expansion whereas the loss of LCRs is more often due to accumulation of amino acid substitutions as opposed to deletions. This dichotomy results in net protein sequence gain over time. We have detected a significant increase in the rate of accumulation of novel LCRs in the ancestral Amniota and mammalian branches, and a reduction in the chicken branch. Alanine and/or glycine-rich LCRs are overrepresented in recently emerged LCR sets from all branches, suggesting that their expansion is better tolerated than for other LCR types. LCRs enriched in positively charged amino acids show the contrary pattern, indicating an important effect of purifying selection in their maintenance.

**Conclusion:**

We have performed the first large-scale study on the evolutionary dynamics of LCRs in protein families. The study has shown that the composition of an LCR is an important determinant of its evolutionary pattern.

## Background

Regions highly enriched in one or a few amino acids, known as low-complexity regions (LCRs), are strikingly abundant in protein sequences [1-3]. For example, 18-20% of human proteins contain at least one single amino acid tandem repeat of size 5 or longer [4,5] and, in eukaryotes, the majority of proteins are more repetitive than expected by chance [6-8]. Many amino acid tandem repeats are likely to originate by replication slippage of triplet repeats in the coding sequence. Replication slippage is a mutational process that expands or contracts microsatellite sequences [9]. However, it has also been noted that some well-conserved amino acid repeats are encoded by heterogeneous DNA repeats [10,11]. These repeats are formed by a combination of different synonymous codons as opposed to repetitive tracts of the same codon. Heterogeneous DNA repeats may have emerged by mechanisms other than slippage or they may be maintained by selective pressure for homogeneous amino acid content [12].

Perfect single amino acid tandem repeats are the best-studied type of LCR. Such tracts are easy to search for in protein sequence libraries (common size cut-offs being 4 or 5 repeat units). They are particularly frequent in transcription factors [5,10,13] and experiments have shown that variations in the length of particular single amino acid repeat tracts, such as glutamine, proline or alanine, can result in changes in the transcriptional activity of the protein containing them [14-16]. They are also of medical interest as a number of neurodegenerative disorders have been shown to be caused by the uncontrolled expansion of glutamine tandem repeats [17], and several developmental diseases are due to formation of unusually long alanine repeats [18]. Potential roles for amino acid repeats in protein evolvability and multifunctionality have also been explored theoretically [12].

The majority of LCRs are more complex than single amino acid tandem repeats [6,19,20], presumably reflecting the combined action of several mutational and selective processes. Functional low complexity regions (LCRs) include long histidine-rich stretches shown to be important for the localization of proteins in nuclear speckles [21], SR-rich domains involved in RNA interactions in splicing factors [22] and charged amino acid regions capable of modulating the activity of several transcription factors [23,24]. LCRs are frequently found in regions of protein disorder [19,25] and the level of repeat perfection correlates with their tendency to be unstructured [26].

We have previously found that, in alignments of orthologous mammalian proteins, both amino acid tandem repeats and LCRs are more often associated with insertions than with deletions [27], suggesting that repetitive regions have a greater tendency to expand than to contract. The accumulation of certain LCRs over time will depend on the intrinsic rate of slippage of the triplets encoding the underlying amino acid repeats, as well as the strength and mode of selection acting on the LCRs. Among triplet repeats, (CAG/GTC)_n_ and (CGG/GCC)_n_ sequences are particularly prone to expand by slippage as they form the most stable secondary structures [28]. These triplet repeats can encode several amino acid tandem repeats: poly-alanine (GCA, GCT, GCG, GCC), poly-glutamine (CAG), poly-serine (AGC), poly-leucine (CTG), poly-cysteine (TGC), poly-arginine (CGG, CGC), poly-glycine (GGC) and poly-proline (CCG). Not surprisingly, some of these amino acid tandem repeats, including poly-alanine, poly-glyicine and poly-proline, are very abundant in mammalian proteins [4,5]. However, others are relatively rare (poly-arginine) or nonexistent (poly-cysteine). As strand or frame should not influence slippage mutation rate, selection seems to modulate the frequency of fixation of LCRs of different composition.

A newly gained LCR can follow two evolutionary paths. If the LCR is not functionally relevant *per se*, point mutations will rapidly accumulate and the LCR will progressively degenerate. In this case the repetitive region will act as raw material within which new functional domains can subsequently form [3]. If, instead, it is functionally relevant, its repetitive nature will tend to be preserved by purifying selection [29,30]. In general, the turnover of amino acid repetitive regions is very high [5,19,31-33], suggesting that they often evolve under very relaxed constraints. However, the higher conservation of human coding repeats compared to those that are non-coding and of similar composition indicates that purifying selection acts on a significant fraction of coding repeats [34].

To improve our understanding of the constraints acting on different types of LCRs, and the variations exhibited by different lineages, we have compared the LCRs present in a large set of chordate homologous protein families. The study has provided an improved global picture of the contribution of LCRs to the evolution of modern proteins.

## Results

### Identification of LCRs in chordates

In order to study the evolutionary dynamics of low-complexity regions (LCRs) in chordates we obtained a large set of homologous genes from five genomes: *Homo sapiens* (human), *Mus musculus* (mouse), *Gallus gallus* (chicken), *Danio rerio* (zebrafish) and *Ciona intestinalis*, which clustered into 4,227 protein families using information from Ensembl Compara [35]. About half of the protein families contained only one gene per species and the remaining ones contained more than one gene in one or several species. This is because we considered 1 to 1 as well as 1 to many orthology relationships as defined in Ensembl, which means that we included genes that had duplicated in particular vertebrate lineages.

We next identified low-complexity regions (LCRs) in the protein sequences with the program SEG (Wootton & Federhen, 1994) using optimized parameter settings for the detection of highly significant repetitive sequences (see details in Methods). The LCRs were, on average, 22 amino acids long, and typically strongly enriched in one or two amino acids (Additional file 1). For comparison, we also identified all perfect single amino acid tandem repeats (ATRs) of size 5 or longer. The LCRs included 31.8% of the ATRs of size 5 amino acids or longer and 98.16% of those of size 10 or longer. The relatively low proportion of short ATRs included in the LCRs reflected the fact that short ATRs did not qualify as LCRs unless they were embedded in larger repetitive regions. Long repetitive regions are more likely to be functionally relevant than short ones, in addition to being more amenable to comparisons between distant species. Mammalian proteins contained significantly more LCRs than chicken or zebrafish proteins (Test of Equal Proportions p < 10^-3^), and the fraction of proteins with LCRs was remarkably small in *C.intestinalis* in comparison with other species (Table 1).

**Table 1:** Number of low-complexity regions (LCRs) in chordate homologous proteins

	**High LCR content**		**Intermediate LCR content**		**Low LCR content**
	**Human**	**Mouse**	**Chicken**	**Zebrafish**	***C. intestinalis***
N proteins	5,518	5,548	5,546	6,820	5,011
N LCRs	753	715	480	666	188
Proteins with LCRs^a^	561 (10.2%)	532 (9.6%)	364 (6.6%)	498 (7.3%)	162 (3.2%)
LCRs per protein^b^	1.34	1.34	1.32	1.34	1.16

^a^Differences were highly significant between species from the groups High LCR content, Intermediate LCR content and Low LCR content (p < 10^-3^). ^b^LCRs per protein was only calculated for proteins with LCRs.

### Structure and function of chordate LCRs

To learn about the compositional structure and functional associations of LCRs we obtained a set of 1,690 non-redundant LCRs by taking one representative LCR sequence when the LCR was conserved in two or more species (see Methods). The majority (71.5%) of LCRs in this non-redundant set were enriched in a single amino acid (defined as the frequency of the most abundant amino acid being more than twice the frequency of any other amino acid) (Table 2). In addition, the majority of proteins in this set (77.34%) included a single amino acid tandem repeat (ATR) of size 5 or longer.

**Table 2:** Most frequent combinations of amino acids in LCRs enriched in one and two residues

	**Enriched in one amino acid**			**Enriched in two amino acids**			
	**LCRs**	**ATRs**	**ATRs/LCRs**		**LCRs**	**SPRs**	**SPRs/LCRs**
E	230	245	1.07	ED	54	8	0.15
S	191	166	0.87	QP	34	8	0.24
G	160	142	0.89	SR	31	30	0.97
P	151	167	1.11	AG	30	1	0.03
A	132	123	0.93	KE	28	17	0.61
Q	107	125	1.17	PG	25	18	0.72
K	78	51	0.65	RG	23	15	0.65
L	42	37	0.88	AP	22	5	0.23
D	30	31	1.03	SG	20	4	0.2
T	28	37	1.32	ER	19	17	0.89
H	27	30	1.11	LP	12	1	0.08
R	22	7	0.32	SE	11	0	0
total	1209	1168	0.95	total	449	190	0.4

*ATRs* single amino acid tandem repeats, of size 5 or longer. *SPRs* short period repeats, of size 3 or longer repeat units.

LCRs within this non-redundant set that were enriched in two amino acids (defined as the frequency of the second most abundant amino acid being more than half the frequency of the most abundant amino acid) constituted 26.57% of LCRs identified in this set of proteins. The set of amino acids forming these two amino acid enriched repeats was similar to the amino acid set forming single amino acid repeats (Table 2). About 40% of these LCRs included tandem repeated units containing two or more different amino acids (short period repeats, SPRs). We found evidence of hexanucleotide slippage for 31.7% of these LCRs: Sequences including at least three tandem two amino acid repeat units (e.g. SRSRSR), consistent with hexanucleotide slippage, were strongly enriched in SR, ER, PG and RG motifs. The remaining LCRs enriched in two amino acids were more chaotically arranged (e.g. AGGAAGGAG). In these latter tracts, we observed that one single nucleotide mutation was usually sufficient to replace one amino acid by the other (Additional file 2: Table S1). The patterns were consistent with the combined action of recent slippage and point mutation events.

We performed Gene Ontology enrichment analysis of proteins containing LCRs dominated by one or by two amino acids (Table 3). As reported in previous studies focusing on amino acid tandem repeats [5], alanine and glutamine were strongly associated with transcriptional regulation functions (Fisher’s Exact test; p < 10^-3^). In fact, there is experimental evidence that such repetitive tracts can have an active role in modulating gene transcription [14,15]. SR-rich LCRs were significantly associated with RNA splicing, in agreement with the presence of SR-domains in many splicing factors [22].

**Table 3:** Overrepresented Gene Ontology terms in human proteins containing particular LCR types

**LCR**	**Gene Ontology term**	**Observed(%)**	**Total (%)**	**p-value**
A	cell cycle	7 (10.77)	691 (2.22)	0,00062
A	regulation of transcription	14 (21.54)	2,208 (7.1)	0,00016
G	RNA splicing	5 (7.58)	332 (1.1)	0,00085
P	actin cytoskeleton organization	9 (15.52)	218 (0.83)	<0,00001
Q	regulation of transcription, DNA-dependent	19 (38)	3,841 (15.2)	0,00007
ED	nucleosome assembly	6 (16.67)	360 (1.93)	0,00006
SR	RNA splicing	8 (61.54)	329 (2.42)	<0,00001

Total refers to all human proteins in Ensembl. Results are for the Biological Process ontology with p < 0.001.

### Formation of novel LCRs

**Figure 1:**
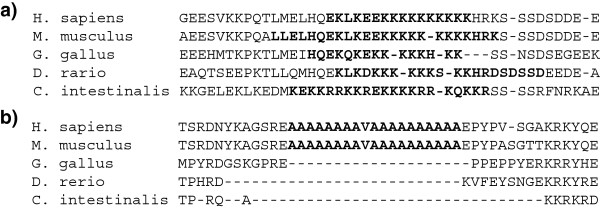
**Conservation of low-complexity regions (LCRs) in chordate homologous protein families.** LCRs are indicated in red. Only the region of the alignment containing the LCR is shown. **a**) Example of conserved chordate LCR enriched in lysines. Corresponds to methionyl aminopeptidase 2 (Ensembl Protein Identifier ENSP00000325312 in humans). **b**) Example of mammalian-specific LCR enriched in alanines. Corresponds to alkylation repair 5 (Ensembl Protein Identifier ENSP00000261650 in humans).

We next determined the phylogenetic branch at which the LCRs had originated. From the initial set of LCRs we discarded those LCRs that were embedded in protein regions that were missing in any of the species, as this mainly reflected incomplete gene annotations or non-conserved exons, rather than specific gain or loss of LCRs. The filtering resulted in 1,158 non-redundant LCRs (Additional file 1). To establish the phylogenetic breadth of these LCRs we employed slightly more relaxed SEG parameters than for the initial LCR definition, as this increased our sensitivity (capacity to detect the LCR in distant species) without compromising specificity (the LCRs were defined with the initial strict criteria in at least one of the species). LCRs conserved in all the species showed a strong overlap in the protein alignment and, by definition, enrichment in the same amino acid/s (Figure 1a). Lineage- or species-specific LCRs were those detected in a subset of species, typically aligning with long gappy regions in other species (Figure 1b), reflecting their origin as insertions.

**Figure 2:**
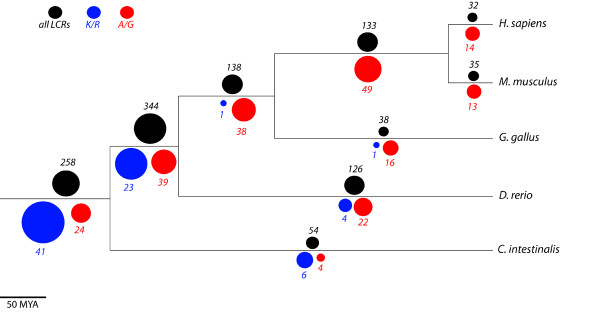
**Relative abundance of low-complexity regions (LCRs) at different phylogenetic depths.** The area of the circles is proportional to LCR relative frequencies. The number of LCRs at each branch is indicated. In black, data for all LCRs. In blue, data for LCRs enriched in positively charged amino acids (K and R). In red, data for LCRs enriched in alanine (A), glycine (G) or both. The LCR phylogenetic distribution of LCRs labeled in blue, and of LCRs labeled in red, deviated significantly from the expected one considering all LCRs (Fisher’s exact test p < 10^-5^).

The distribution of LCRs in different branches of the vertebrate phylogeny is plotted in Figure 2. When compared to the expected rate of LCR accumulation according to the length of the branches in million year (Mya) units, using paleontological dates for the different nodes [36], we detected a significant excess of LCRs in the common mammalian and chicken branch (Amniota) and a significant lack of LCRs in the chicken and *C.intestinalis* branches (Additional file 2: Table S2, p < 10^-5^). The estimated rate of formation of LCRs in the Amniota branch, spanning from approximately 312 to 416 Mya ago, was 1.33 LCRs/Mya. The rate decreased in the mammalian, human and mouse branches, to about 0.52-0.57 LCRs/Mya. The slow down in the chicken branch was even more marked, with only 0.12 novel LCRs being gained each Mya on average.

**Figure 3:**
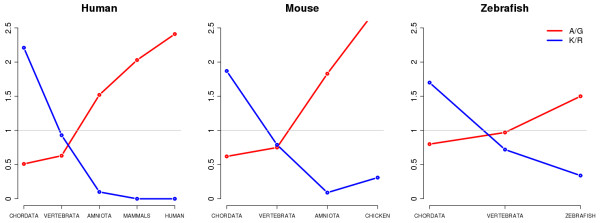
**Observed versus expected number of LCRs in the different branches leading to an extant organism.** Data is shown for LCRs enriched in K or R (in blue) and in A, G or AG (in red). The observed distribution of LCRs labeled in blue deviated significantly from the expected one in all three cases (Fisher’s exact test p < 10^-3^). The observed distribution of LCRs labeled in red deviated significantly from the expected one for human LCRs (Fisher’s exact test p < 10^-3^).

Does LCR composition influence the LCR’s degree of conservation? We observed that LCRs enriched in alanine (A) and/or glycine (G) were progressively more abundant in younger LCR sets (Figure 2 and Additional file 2: Table S3). The two together represented about 50% of the human and mouse-specific LCRs, 35% of the Amniota-specific LCRs and 11% of the LCRs conserved in all chordate species. This trend was consistent across the four different vertebrate branches (Figure 3 and Additional file 2: Table S4, mouse is very similar to human and thus not shown). The other two types of LCRs that showed significant deviations were lysine (K) and arginine (R)-rich LCRs. In this case the opposite trend was observed, as a larger than expected proportion of them were highly conserved (Figure 2 and Figure 3, p < 10^-3^).

### Dynamics of LCR gain and loss

The level of conservation of LCRs allowed us to estimate the number of LCRs gained in different vertebrate branches but, in order to truly reconstruct LCR history, we also needed to estimate the rate of LCR losses. This was achieved by comparing the number of ancestral LCRs conserved in different species to those that were missing in one particular branch (see Methods). We estimated that, depending on the branch, between 10% and 25% of the ancestral LCRs had been lost. In order to understand whether LCR loss was due to sequence divergence of the LCR tract (beyond recognition by SEG) or to deletion of all or most of the LCR region we examined the region where the LCR had been lost, if it was very gappy (>50% gaps) we assumed the repetitive tract had been deleted. Interestingly, the majority of LCRs appeared to have been lost because of sequence divergence as opposed to deletion (Additional file 2: Table S5). This contrasts with the fact that LCRs seem to emerge through insertion rather than sequence divergence, since lineage-specific LCRs typically mapped to gappy regions in the homologous proteins (e.g. 75% of the human-specific LCRs mapped to gappy regions in the proteins from all the other species, whereas only 17% of the LCRs annotated as lost in the human branch mapped to gappy regions in the human protein). These results indicate that LCRs more frequently expand than contract, and thus, contribute to extending protein sequences.

**Figure 4:**
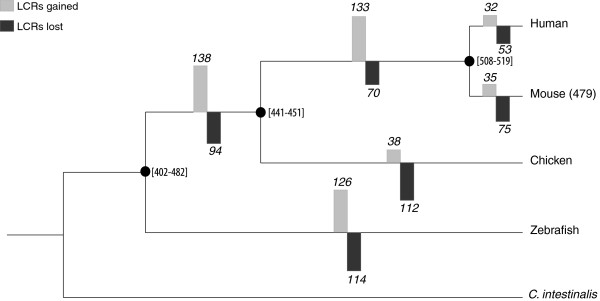
**Dynamics of gain and loss of low-complexity regions (LCRs) in vertebrate homologous protein families.** The LCRs gained in each branch correspond to LCRs observed at different phylogenetic depths. The LCRs lost are estimated from LCR phylogenetic distribution data (see Methods). Values in square brackets in internal nodes represent the estimated number of ancestral LCRs.

By combining the data on LCR gains and loses we estimated the number of LCRs at the ancestral nodes (in square brackets in Figure 4, see Methods for more details). In both the Amniota and mammalian branches the LCRs gained clearly outnumbered the LCRs lost, resulting in a net LCR increase during this period. In the chicken branch the number of LCR losses was comparable to that of other branches, but the number of LCR gains was lower, indicating reduced rates of slippage or stronger purifying selection against LCRs.

## Discussion

The results of this study provide novel insights into the role of low-complexity regions (LCRs) in protein evolution and function. The use of homologous protein families has allowed us to compare the same proteins in different organisms, and thus focus on lineage and age-related features on LCR evolution because we are able to control for the differences among genes, which affected previous comparisons [4,13]. By analyzing 4,227 protein families, we have identified about 402 LCRs that have been formed since the vertebrate common ancestor in different vertebrate lineages (Figure 4). These LCRs have provided abundant material for new functions to arise and they have generally increased the lengths of these proteins. The accumulation of LCRs has not been even with respect to the different vertebrate lineages and we have detected an important net gain of LCRs in the Amniota and mammalian ancestral branches.

In the initial homologous protein set, about 10% of the human and mouse proteins contain at least one LCR, compared to about 7% in chicken and zebrafish. Part of this difference is due to a fraction of mammalian LCRs that are embedded in regions of proteins that are lineage-specific or that represent alternatively spliced exons. Consistent with this, alternatively spliced exons have recently been shown to be enriched in amino acid repeats [37]. However, when we discard such regions, mammalian species still contain more LCRs than the other chordate species analysed (the percentage of proteins with at least one LCR being about 9% in humans and mouse, compared to 7% in chicken and in zebrafish). We have been able to determine that this is due to an increased rate of net LCR gain in the Amniota and mammalian ancestral branches, a tendency that appears to be reversed in the chicken-specific branch. Although zebrafish and chicken contain a similar fraction of proteins with LCRs, 25% of zebrafish LCRs are species-specific whereas only 10% of chicken LCRs are species-specific. Zebrafish is the species with more paralogous genes in the protein families studied here, so relaxed selection following gene duplication may have increased the accumulation of LCRs in this lineage. In *C.intestinalis* the percentage of proteins with LCRs is much lower (3%) than in the vertebrate species (Table 1, Figure 2). The reason for this is unclear because this species is the outgroup in the estimation of branch-specific LCR loses and gains, and therefore we have not been able to estimate the rates of these two types of events.

Many, but not all, the amino acids found within LCRs are abundant in proteins in general (Additional file 2: Table S6 and Figure S1). These includes several amino acids associated with a low synthesis cost, such as alanine, glycine, proline serine and glutamic acid [38]. In contrast, leucine, which is also very common within proteins in general, is not particularly frequent in LCRs. High codon expandability can favor the formation of repeats of amino acids that are *per se* not abundant in proteomes, such as glutamine. For example CAG/CTG triplets show a high propensity to undergo slippage [39], which explains why glutamine tandem repeats are the longest on average in various species [5,40,41].

Are LCRs involved in protein function? A Gene Ontology term enrichment test detected known associations such as an overrepresentation of serine and arginine (SR)-rich LCRs in proteins involved in RNA splicing [22], and alanine- and glutamine-rich LCRs in transcription factors [5,13]. We have also found an association between proline-rich LCRs and proteins involved in actin cytoskeleton organization. One of these proteins is dynamin, which is a GTPase involved in cytokinesis. The proline-rich region within this protein has been reported to act as a microtubule-binding domain [42]. We have also found that there is an enrichment in acidic stretches in proteins involved in nucleosome assembly.

An important novel finding in the present study is that the composition of LCRs that have arisen more recently differs from the composition of LCRs that have a more ancient origin. The subset of relatively young LCRs are enriched in alanine and glycine-rich LCRs, whereas the subset of older LCRs are enriched in lysine and arginine-rich LCRs. This is very interesting because it has been suggested that alanine and glycine were probably the first amino acids to appear on earth, forming poly-alanine and poly-glycine oligomers [43,44]. Along the same lines, trinucleotide slippage has been proposed to be an important mechanism for the formation of the first peptides [45]. The fact that young protein coding genes in today’s genomes (for example mammalian-specific genes) are enriched in LCRs, suggests that slippage has been important for the formation of novel proteins in recent times [46].

Mutations that generate novel repetitive sequences, or extend them, may become fixed in a population provided they are not deleterious. Alanine and glycine are small amino acids with a low impact on protein structure or function compared to larger charged or aromatic amino acids. In addition, these amino acids are encoded by triplets prone to expand by slippage [28]. These factors probably favour their initial fixation by genetic drift. Prior evidence suggests that some alanine tandem repeats can influence the transcriptional activity of the proteins harbouring them [14,47]. If they do not acquire a novel function, these sequences may diverge beyond recognition as an LCR. In contrast, lysine and arginine-rich LCRs are only frequent in the set of highly conserved LCRs. One of the codons for arginine, CGC, is prone to slippage, so purifying selection possibly acts to prevent the fixation of arginine-rich LCRs in the species examined. In particular contexts, however, these tracts are probably beneficial and become fixed by positive selection. Subsequently, purifying selection may contribute to the maintenance of these LCRs. Other frequently occurring LCRs, such as those enriched in glutamic acid, proline or serine, show intermediate distribution patterns, indicating that they are relatively well tolerated in proteins.

It has been argued that selective pressure for increased G + C content in mammalian genomes could have triggered the expansion of alanine, glycine and proline repeats, all of which are encoded by CG-rich codons [48,49]. Analyses of mammalian genomes has shown that genes encoding amino acid tandem repeats tend to have higher GC content than the other genes [5]. A positive correlation between coding sequence GC content and amino acid tandem repeat frequency has also been found in plants and fungi, but not in fruit flies [50]. In addition, some eukaryotic species with AT-rich genomes contain a very high number of protein low-complexity regions [51], indicating that the relationship between LCR accumulation in vertebrates and overall genomic GC content or GC heterogeneity is not universal. In our study, zebrafish and *C.intestinalis* genomes have a lower and more homogeneous GC content than chicken or mammalian genomes but the number of LCRs per protein in zebrafish and chicken is comparable. We have found that sequences encoding alanine-rich LCRs, enriched in GCX codons, are comparatively more abundant in mammals and chicken, but those encoding glycine-rich and proline-rich LCRs, enriched in GGX and CCX codons, respectively, are similarly abundant in all species. Therefore it appears that the relationship between genomic overall GC content or GC heterogeneity and LCR accumulation in vertebrates is more complex than previously reported.

## Conclusions

The study presented here is the first attempt to follow the evolutionary history of a large set of LCRs in order to obtain novel insights into the role of LCRs in protein evolution. It uncovers important differences in the rate of LCR gain in different vertebrate lineages and identifies novel LCR functional associations. It also shows that the composition of an LCR plays a role in determining its evolutionary dynamics, likely because it affects both the rate of slippage as well as the strength and mode of selection.

## Methods

### Sequence datasets and alignment

*Homo sapiens* (human), *Mus musculus* (mouse), *Gallus gallus* (chicken), *Dario rerio* (zebrafish) and *Ciona intestinalis* one:one, apparent one:one and one:many orthologous genes were obtained from Ensembl v.56 using BioMart [52]. We used releases GRCh37 (*Homo sapiens*), NCBIM37 (*Mus musculus*), WASHUC2 (*Gallus gallus*), Zv8 (*Dario rerio*) and JGI2 (*Ciona intestinalis*). By combining the orthology information for the different species, we obtained a set of 4,227 homologous gene families that had at least one gene encoding a protein longer than 60 amino acids in each of the five species. The average number of genes per family was seven. Half of the families contained one gene per species (2,110 families). About 25% of the families contained several genes from the same species, reflecting recent, species-specific, duplications. The remaining families contained multiple genes in different species, as a result of older duplications (occurring at the vertebrate, *Amniota* or *Euarchontoglires* branches) or because of several species-specific independent duplications.

For each family we built protein sequence multiple alignments with T-Coffee [53]. When more than one protein sequence per gene was available, we took the longest one. The final dataset for analysis consisted of 5,518 human proteins, 5,548 mouse proteins, 5,546 chicken proteins, 6,820 zebrafish proteins and 5,011 *C.intestinalis* proteins.

### Identification of LCRs

We defined low-complexity regions (LCRs) using SEG [2], which divides sequences into segments of low- and high-complexity. The parameters used were window = 15, K_1_ = 1.5 and K_2_ = 1.8. These parameters ensured that the regions identified corresponded to strongly compositionally biased sequences while at the same time allowing for substantial sequence diversity. Low-complexity segments defined by the SEG algorithm represent compositionally biased regions based only on residue composition and taking into account the improbability of appearance of such sequences. We identified 753 LCRs in human, 715 in mouse, 480 in chicken, 666 in zebrafish and 188 in *C.intestinalis*.

### Compositional analysis of LCRs

We developed several in-house Perl programs to analyze the LCRs. For each LCR, we stored the different amino acid frequencies, the tandem repeats of single amino acids of size 5 repeat units or longer (amino acid tandem repeats, ATRs) and the tandem repeats of repeat units formed by 2 or more different aminoacids of size 3 repeat units or longer (short period repeats, SPRs). LCRs enriched in one amino acid were defined as those in which the frequency of the most abundant amino acid was more than twice the frequency of any other amino acid. LCRs similarly enriched in two amino acids were those in which the frequency of the second most abundant amino acid was more than half the frequency of the most abundant amino acid. Using the same definition, we also obtained regions enriched in more than two amino acids.

To build a non-redundant set of LCRs we used a Perl script to identify all LCRs that overlapped in the alignments and showed the same amino acid enrichment (for example enriched in A, or in ED), and to select the longest one of them as representative. Using these criteria, we obtained 1,690 low-complexity regions: 1,209 LCRs enriched in one amino acid, 449 LCRs enriched in two amino acids and 32 LCRs enriched in more than two amino acids.

For the most common combinations of two amino acids (Table 2) we investigated if the corresponding codons showed high or low sequence similarity (Additional file 2: Table S1). The high similarity group was formed by cases in which all codons for the first amino acid were only one nucleotide mutation away from codons for the second amino acid, and viceversa (for example E and D, the first amino acid is encoded by GAA and GAG and the second amino acid by GAT and GAC). The group with no similarity corresponded to cases for which one nucleotide mutation was not sufficient to replace one amino acid by the other (for example E and R, as R is encoded by AGA and AGG). The group with intermediate similarity was formed by cases in which only some codon pairs were highly similar.

### Identification of conserved LCRs

We used an in-house Perl program to determine the degree of phylogenetic conservation of LCRs using parsimony criteria. The depth of LCR conservation was defined on the basis of the species in which the LCR was present, using LCR sequence overlap as evidence of conservation. If one species had several paralogous gene copies, the presence of the LCR in one of the copies was considered sufficient to tag the species as containing the LCR. To avoid underestimating the age of LCRs we also considered LCRs identified by SEG using more relaxed parameters (window = 15, K_1_ = 1.9 and K_2_ = 2.3) in the definition of a LCR’s phylogenetic breadth. Some LCRs were embedded in longer amino acid sequences that had no counterparts in one or more homologous proteins from other species. These LCRs were discarded, as they could not be properly compared across species. To do so, we first defined LCR containing regions as sequences that extended 25 amino acids at each side of the LCR. We subsequently filtered out all LCR containing regions that aligned with regions with > 95% gaps in any of the homologous proteins from other species. The final dataset comprised 1,158 LCRs present in one or more homologous proteins from different species. By species we obtained 487 LCRs in human, 479 LCRs in mouse, 367 LCRs in chicken, 494 LCRs in zebrafish and 167 LCRs in *C.intestinalis*.

To determine the origin of each LCR we inspected the range of species in which the LCR was found. In the case of gaps in the phylogenetic distribution of an LCR we considered the most parsimonious explanation of the LCR having been lost in one or more lineages than having been independently gained in several lineages. Thus, for an LCR to be classified as Chordata we required it to be present in *C.intestinalis* and at least one other species (although the majority of them were present in all or nearly all species). Similarly, Vertebrate LCRs were defined as those present in zebrafish and at least one other species out of chicken, mouse and human (but not in *C.intestinalis*). LCRs classified as Amniota were present in chicken and in at least one mammalian species, but absent from *C.intestinalis* and zebrafish. Mammalian LCRs were those present in human and mouse but not in other species. Finally, if a LCR was present in only one species, we considered it was species-specific. The percentage of species-specific LCRs that mapped to regions with more than 50% of gaps in all other species was 74.3% for human-specific LCRs, 75.7% for mouse-specific LCRs, 46% for chicken-specific LCRs, 39% for zebrafish-specific LCRs and 34% for *C.intestinalis*-specific LCRs. We classified 1,104 LCRs in different age groups. The LCRs from a given phylogenetic group were considered to have been formed in the branch preceding the common ancestor of the group.

### Estimation of LCR lost rate and number of LCRs in ancestral nodes

As already mentioned, in some cases the LCR was missing from one or more species. We identified two types of loss of ancestral LCRs: deletion of the LCR – when the percentage of gaps in the corresponding aligned region was > 50% - and increase in sequence complexity – when gaps ≤ 50%.

The rate of LCR loss in branch *X* was calculated as follows:

$$r_{X}=L_{X}/\left(L_{X}+C_{X}\right),$$

where *L*_*X*_ is the number of ancestral LCRs lost in branch *X* and *C*_*X*_ the number of ancestral LCRs conserved in branch *X*.

For example, *L*_*zebrafish*_ will be the LCRs found in *C.intestinalis* and in chicken but not in zebrafish, and *C*_*zebrafish*_ the ones found in *C.intestinalis*, chicken and zebrafish.

The number of LCRs in an ancestral node *a* was then calculated as:

$$n_{a}=\left(N_{d}-Z_{d}\right)/\left(1-r_{a-d}\right),$$

where *N*_*d*_ is the number of LCRs in a derived node *d*, *Z*_*d*_ the number of LCRs gained in the derived node *d*, and *r*_*a*−*d*_ the rate of LCR loss in the branch connecting *a* and *d*.

For example, in the case of the mammalian ancestor (*n*_*mammal*_) we estimated 508 LCRs using the human branch and 519 LCRs using the mouse branch. In the first case *N*_*human*_= 487, *Z*_*human*_= 32, and *r*_*mammal*−*human*_ =0.104, and in the second case *N*_*mouse*_=479, *Z*_*mouse*_=35 and *r*_*mammal*−*mouse*_ =0.145. Using several derived branches provides value intervals for the estimations of the number of ancestral LCRs, for example 508–519 in the case of the mammalian ancestral node.

### Gene ontology annotation

We extracted all Gene Ontology (GO) terms [54], for Biological Process, Molecular Function and Cellular Component, for all human proteins using Biomart at Ensembl [52]. The frequency of occurrence of different GO identifiers was calculated for the complete genome and for proteins containing LCRs enriched in different amino acids. We tested the hypothesis of whether the frequency of occurrence of different GO terms was higher than expected in proteins containing particular LCR types (Fisher test, p-value < 10^-3^). We only considered cases in which the GO term occurred at least five times in the subset of interest. Highly redundant GO terms were not considered.

### Statistical tests and graphics

All statistical analyses were performed with the R statistical package [55]. To test for differences in the proportion of proteins with LCRs in different species we used the test of equal proportions. To identify LCRs overrepresented in proteins annotated with particular GO terms we used the Fisher exact test. To test for differences between the number of observed and expected LCRs in different categories we used the chi-square or the Fisher exact test.

## Authors' contributions

NR-T performed the analyses and wrote a first draft of the manuscript. MMA designed the study and wrote the final version of the manuscript. All authors read and approved the final manuscript.

## Supplementary Material

Additional file 1 Is an excel file containing a list of LCRs per species, a list of non-redundant LCRs, and a list of LCRs conserved at different phylogenetic depths. Click here for file

Additional file 2 Contains 6 Tables and 1 Figure as supplementary material. Click here for file
