# Supplementary material for: Dissecting the role of low-complexity regions in the evolution of vertebrate proteins
Source: BMC Evol Biol. 2012 Aug 24;12:155. doi: 10.1186/1471-2148-12-155 (PMC3523016; doi:10.1186/1471-2148-12-155)
Supplement: Additional file 2 — Contains 6 Tables and 1 Figure as supplementary material. [file 1471-2148-12-155-S2.pdf]

Radó-Trilla and Albà (2012), additional data file 2.

Table 1

Level of codon similarity	Amino acid pairs
High similarity	ED, AG, KE, AP, RG
Intermediate similarity	QP, SR, SG, LP
No similarity	PG, ER, SE

Table 1. Classification of amino acid pairs based on codon similarity. High similarity is when one point mutation in any of the codons of the first amino acid can result in a codon for the second amino acid. Intermediate similarity is when one point mutation can led to the second amino acid for only some of the codons. No similarity is when one point mutation is not sufficient to go from the first amino acid to the second amino acid.

Table 2

Branch	LCRs (obs)	Mya	LCRs(obs)/Mya	LCRs (exp)	p-value
amniota	138	104	1.33	43	1.19×10^{-14}
mammals	133	251	0.53	104	0.0451
human	32	61.5	0.52	26	0.4988
mouse	35	61.5	0.57	26	0.2906
chicken	38	312	0.12	130	1.43×10^{-14}
zebrafish	126	416	0.3	173	0.0015

Table 2. LCRs gained in different branches of the vertebrate phylogeny. Mya: estimated length of the branch in million years (Mya), see text for more details. p-value is from a test of equal proportions between LCRs observed (obs) and LCRs expected (exp) given branch lengths. Differences between LCRs observed and LCRs expected were significant by a chi-square test ($p<10^{-5}$).

Table 3

Observed

	Chordata	Vertebrata	Amniota	Mammals	Human	Mouse	Chicken	Zebrafish	Ciona	Total
E	32	52	19	12	3	3	3	23	2	149
S	23	47	6	9	2	1	2	25	7	122
G	18	22	17	19	6	6	1	14	3	106
P	23	24	17	12	2	1	2	6	0	87
A	5	10	15	26	7	6	12	8	1	90
Q	11	19	11	7	2	5	1	7	11	74
K	29	16	1	0	0	0	1	3	6	56
D	8	6	0	0	0	1	1	2	1	19
R	12	7	0	0	0	0	0	1	0	20
ED	16	17	3	0	0	1	0	1	1	39
SR	16	10	1	0	0	0	0	1	1	29
AG	1	7	6	4	1	1	3	0	0	23
PG	3	11	3	3	1	0	2	0	1	23
RG	7	6	0	0	0	0	3	3	2	20
AP	2	3	2	3	1	1	3	2	0	16
SG	0	4	1	3	2	0	0	2	0	12
ER	3	8	0	0	0	0	0	0	2	13
LP	0	1	1	4	0	0	0	0	0	6
SE	3	1	0	0	0	0	0	1	0	5
QP	0	6	8	5	0	1	0	0	0	20
others	46	67	27	26	5	8	7	27	16	167
Total	258	344	138	133	32	35	38	126	54	1096

Expected

	Chordata	Vertebrata	Amniota	Mammals	Human	Mouse	Chicken	Zebrafish	Ciona
E	35	47	19	18	4	5	5	17	7
S	29	38	15	15	4	4	4	14	6
G	25	33	13	13	3	3	4	12	5
P	20	27	11	11	3	3	3	10	4
A	21	28	11	11	3	3	3	10	4
Q	17	23	9	9	2	2	3	9	4
K	13	18	7	7	2	2	2	6	3
D	4	6	2	2	1	1	1	2	1
R	5	6	3	2	1	1	1	2	1
ED	9	12	5	5	1	1	1	4	2
SR	7	9	4	4	1	1	1	3	1
AG	5	7	3	3	1	1	1	3	1
PG	5	7	3	3	1	1	1	3	1
RG	5	6	3	2	1	1	1	2	1

AP	4	5	2	2	0	1	1	2	1
SG	3	4	2	1	0	0	0	1	1
ER	3	4	2	2	0	0	0	1	1
LP	1	2	1	1	0	0	0	1	0
SE	1	2	1	1	0	0	0	1	0
QP	5	6	3	2	1	1	1	2	1
others	39	52	21	20	5	5	6	19	8
Total	258	344	138	133	32	35	38	126	54

Table 3. Observed and expected frequencies of different LCR types conserved at different phylogenetic depths. The data corresponds to Figure 2 in main manuscript file.

Table 4

A,G,AG	Chordata	Vertebrata	Amniota	Mammals	Human
observed	24	39	38	49	14
expected	47	62	25	24	6
observed/expected	0,51	0,63	1,52	2,03	2,41

A,G,AG	Chordata	Vertebrata	Amniota	Chicken
observed	24	39	38	16
expected	39	52	21	6
observed/expected	0,62	0,75	1,83	2,8

A,G,AG	Chordata	Vertebrata	Zebrafish
observed	24	39	22
expected	30	40	15
observed/expected	0,8	0,97	1,5

K,R	Chordata	Vertebrata	Amniota	Mammals	Human
observed	41	23	1	0	0
expected	19	25	10	10	2
observed/expected	2,21	0,93	0,1	0	0

K,R	Chordata	Vertebrata	Amniota	Chicken
observed	41	23	1	1
expected	22	29	12	3
observed/expected	1,87	0,79	0,09	0,31

K,R	Chordata	Vertebrata	Zebrafish
observed	41	23	22
expected	24	32	12
observed/expected	1,7	0,72	0,34

Table 4. **Observed and expected frequencies of different types of LCRs conserved at different phylogenetic depths.** The data corresponds to Figure 3 in main manuscript file.

Table 5

	Loss rate (loss/total)	% LCRs loss by gain of sequence complexity
Human	0.104 (17/164)	83.3 %
Mouse	0.145 (25/172)	71.4 %
Mammal	0.155 (34/134)	68.1 %
Chicken	0.254 (9/38)	70.8 %
Amniota	0.233 (7/30)	89.5 %
Zebrafish	0.237 (16/103)	73.9 %

Table 5. Rate of LCR loss in different branches. % Increase in complexity: percentage of cases of LCR loss due to increase in sequence complexity (beyond recognition of the LCR by SEG, gaps in the LCR aligned region < 50%) and not to LCR deletion.

Table 6

	Observed in proteins					Observed in LCRs				
	human	mouse	chicken	zebrafish	Ciona	human	mouse	chicken	zebrafish	Ciona
**A**	0.0692	0.0692	0.0675	0.0647	0.0593	0.1227	0.1142	0.0848	0.0473	0.0226
**F**	0.0367	0.0367	0.0375	0.0373	0.0406	0.0038	0.0038	0.0043	0.0054	0.0063
**L**	0.0985	0.0988	0.0974	0.0966	0.0913	0.0410	0.0388	0.0306	0.0205	0.0122
**I**	0.0459	0.0448	0.0485	0.0468	0.0553	0.0036	0.0033	0.0046	0.0042	0.0097
**M**	0.0220	0.0222	0.0228	0.0244	0.0254	0.0042	0.0049	0.0062	0.0059	0.0093
**V**	0.0609	0.0619	0.0619	0.0634	0.0663	0.0151	0.0164	0.0122	0.0166	0.0120
**S**	0.0806	0.0815	0.0802	0.0830	0.0800	0.1381	0.1331	0.1450	0.1621	0.1378
**P**	0.0598	0.0591	0.0539	0.0535	0.0464	0.1543	0.1430	0.1227	0.0893	0.0906
**T**	0.0519	0.0523	0.0521	0.0541	0.0583	0.0214	0.0231	0.0198	0.0260	0.0666
**Y**	0.0274	0.0275	0.0291	0.0282	0.0311	0.0032	0.0039	0.0050	0.0057	0.0122
**H**	0.0254	0.0256	0.0252	0.0261	0.0260	0.0210	0.0194	0.0211	0.0342	0.0133
**Q**	0.0472	0.0474	0.0472	0.0473	0.0424	0.0894	0.1021	0.0819	0.0629	0.0863
**N**	0.0375	0.0366	0.0400	0.0393	0.0500	0.0042	0.0045	0.0060	0.0108	0.0330
**K**	0.0612	0.0606	0.0644	0.0608	0.0671	0.0394	0.0416	0.0695	0.0770	0.1211
**D**	0.0507	0.0512	0.0513	0.0536	0.0553	0.0399	0.0409	0.0540	0.0764	0.0628
**E**	0.0731	0.0727	0.0736	0.0711	0.0649	0.1282	0.1398	0.1580	0.1609	0.0820
**C**	0.0211	0.0213	0.0218	0.0218	0.0215	0.0014	0.0030	0.0019	0.0071	0.0005
**W**	0.0118	0.0117	0.0117	0.0115	0.0116	0.0005	0.0007	0.0011	0.0007	0.0007
**R**	0.0555	0.0560	0.0539	0.0558	0.0509	0.0432	0.0459	0.0675	0.0660	0.1046
**G**	0.0634	0.0630	0.0600	0.0606	0.0562	0.1254	0.1177	0.1038	0.1210	0.1166

Table 6. Relative amino acid frequencies in complete proteins and in the LCR fraction. Complete proteins were from orthologous protein families (see main manuscript text). LCRs were identified using SEG parameters window=15, K_1=1.5 and K_2=1.8 in the same set of protein families.

Figure 1

A

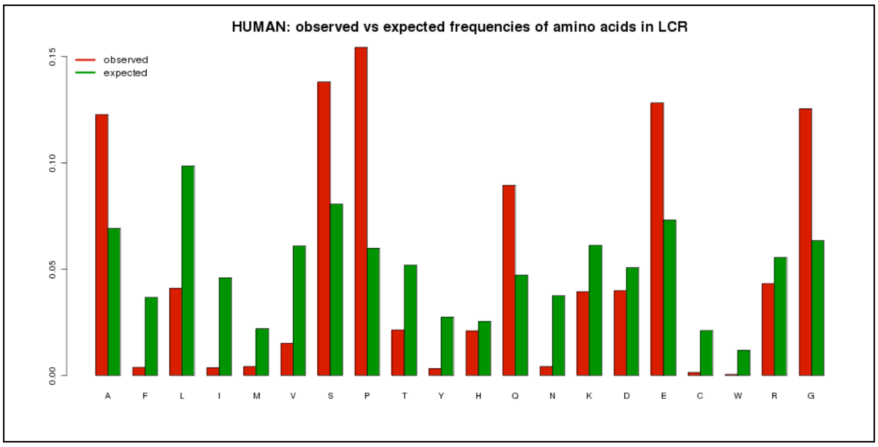

HUMAN: observed vs expected frequencies of amino acids in LCR
observed
expected
0.00 0.05 0.10 0.15
A F L I M V S P T Y H Q N K D E C W R G

B

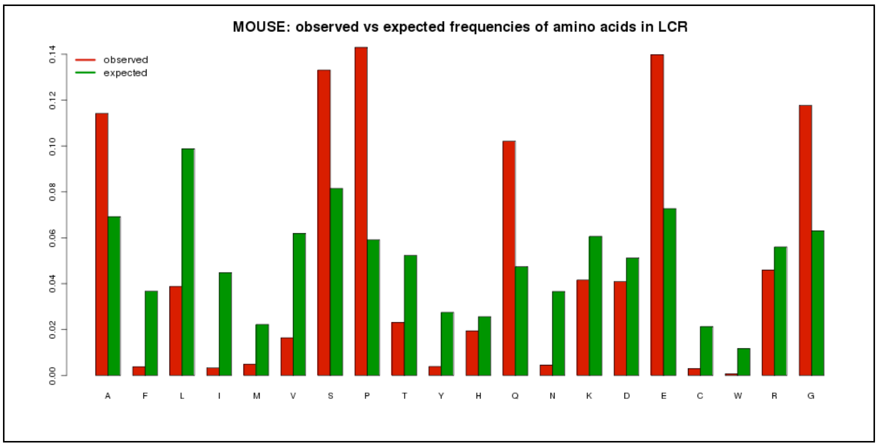

MOUSE: observed vs expected frequencies of amino acids in LCR
observed
expected
0.00 0.02 0.04 0.06 0.08 0.10 0.12 0.14
A F L I M V S P T Y H Q N K D E C W R G

C

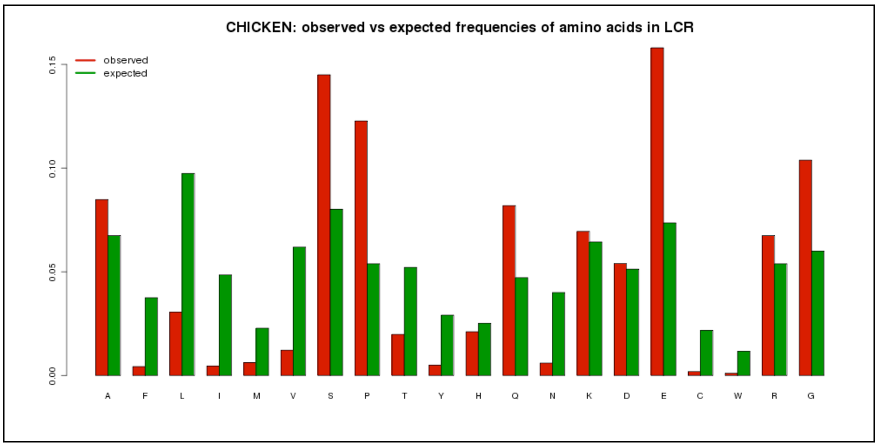

CHICKEN: observed vs expected frequencies of amino acids in LCR
observed
expected
0.00 0.05 0.10 0.15
A F L I M V S P T Y H Q N K D E C W R G

D

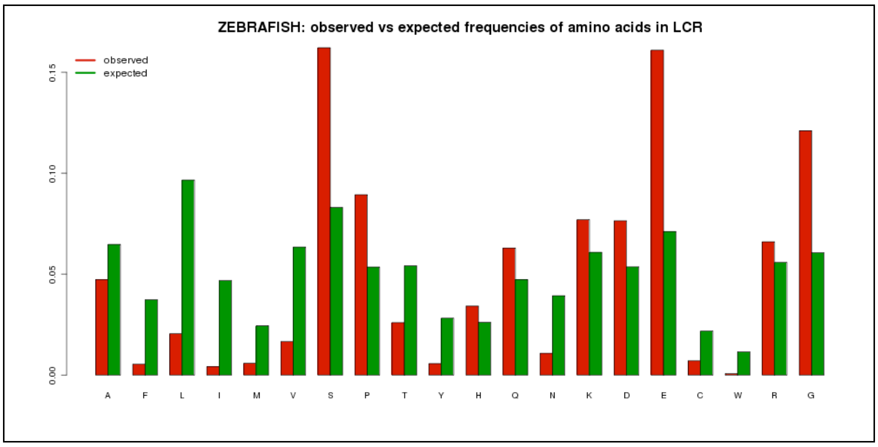

E

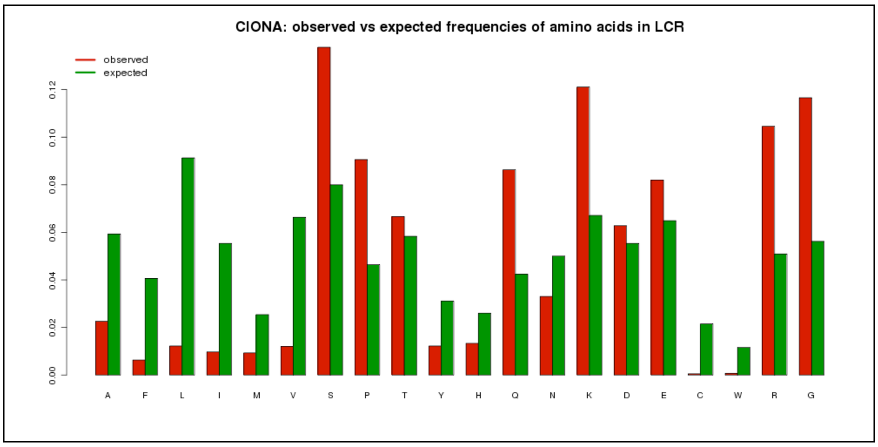

Figure 1. Histogram representation of the relative amino acid frequencies in complete proteins and in the LCR fraction. The data is from Table 6.
